# Effects of Fungicides and Nontarget Pesticides on Accumulation of the Mycotoxin Deoxynivlenol in Wheat

**DOI:** 10.3390/toxics11090768

**Published:** 2023-09-10

**Authors:** Chao Ju, Fan Jiang, Yuan Gao, Tongwu Chen, Jiakuo Cao, Junbo Lv, Yanxiang Zhao, Yongquan Zheng, Wei Guo, Jinguang Huang

**Affiliations:** 1 Shandong Engineering Research Center for Environment-Friendly Agricultural Pest Management, College of Plant Health and Medicine, Qingdao Agricultural University, Qingdao 266109, China; juchao@qau.edu.cn (C.J.); m17860729070@163.com (F.J.); 18660897718@163.com (Y.G.); 13705167186@163.com (T.C.); 17852327371@163.com (J.C.); jblyu@foxmail.com (J.L.); zhaoyx@qau.edu.cn (Y.Z.); zhengyongquan@qau.edu.cn (Y.Z.); 2 Institute of Food Science and Technology, Chinese Academy of Agricultural Sciences/Key Laboratory of Agro-Products Quality and Safety Control in Storage and Transport Process, Ministry of Agriculture and Rural Affairs, Beijing 100193, China

**Keywords:** deoxynivalenol, *Fusarium graminearum*, wheat, H_2_O_2_, pesticides, in vitro, in vivo

## Abstract

Deoxynivalenol (DON) is an important virulence factor of the *Fusarium* head blight of wheat and threatens the health of humans. The effect of fungicides on DON production after stressing wheat to produce H_2_O_2_ and the effect of nontarget pesticides on DON accumulation are largely unknown. Five pesticides were selected to explore the effect of pesticide-induced oxidative stress on DON production in vitro and in vivo. Epoxiconazole and hexaconazole significantly induced an increase in H_2_O_2_ in vitro, and H_2_O_2_ further stimulated the production of DON and the expression of the Tri5 gene. Imidacloprid, isoproturon, and mesosulfuron-methyl had no direct effect in vitro. All pesticides activated the activities of superoxide dismutase, catalase, and peroxidase in wheat and caused the excessive accumulation of H_2_O_2_. However, excessive H_2_O_2_ did not stimulate the accumulation of DON. Imidacloprid indirectly stimulated the production of DON in vivo, which may be due to its impact on the secondary metabolism of wheat. In brief, pesticide-induced H_2_O_2_ in vitro is an important factor in stimulating DON production, but the stressed physiological H_2_O_2_ in wheat is not sufficient to stimulate DON production. The bioaccumulation results indicated that imidacloprid and epoxiconazole increase the risk of DON contamination, especially under field spraying conditions.

## 1. Introduction

*Fusarium* head blight (FHB), which is mainly caused by *Fusarium graminearum*, is a devastating disease in wheat (*Triticum aestivum* L.) [1]. In addition to causing a serious loss of wheat yield, this pathogen can also produce a variety of trichothecene mycotoxins during infection, such as deoxynivalenol (DON), acetyl deoxynivalenol (15-ADON and 3-ADON), and nivalenol [2]. Among these mycotoxins, DON has been confirmed to be the dominant and most common mycotoxin, and can reduce the defense mechanisms of wheat plants and aggravate FHB epidemics [2,3,4]. Because DON can inhibit DNA, RNA, and protein synthesis and impair the immune response of humans and animals [5], the excessive consumption of DON-contaminated wheat can cause symptoms such as vomiting, diarrhea, anemia, growth retardation, and even spermatogenesis disorders [6,7]. The FAO and WHO also list DON as one of the most dangerous natural food contaminants [8]. In China, the relative contamination of wheat by DON and its products in different regions is much higher than that of other mycotoxins, with detection rates ranging from 58.7% to 97.2%, exceeding rates (>1000 μg kg^−1^) ranging from 4.6% to 70%, and maximum detection concentrations reaching 3030 μg kg^−1^ to 56,100 μg kg^−1^ [9,10,11,12,13]. In addition, DON has a long residual period because of its stability, which poses a greater threat to food safety and human health [14].

There are many external factors affecting DON biosynthesis, such as nutrient sources, pH, and light [2]. However, one of the inevitable factors is the use of fungicides. The application of fungicides to control FHB is the main way to control DON. However, some studies have shown that although fungicides can prevent the spread of FHB, they cannot always reduce the accumulation of DON [9,15]. Some fungicides, such as carbendazim, epoxiconazole, and azoxystrobin, can significantly induce the biosynthesis of DON at sublethal concentrations [16,17,18]. These different fungicides have a common inducing factor, that is, the increase in oxidative stress induced by fungicides, which is manifested by the excessive release of H_2_O_2_ in *F. graminearum* [16,17,18]. Audenaert et al. also observed that prothioconazole can stress *F. graminearum* to produce H_2_O_2_, which can further stimulate the biosynthesis of DON [19]. Ponts et al. verified that the exogenous application of H_2_O_2_ can directly trigger *F. graminearum* to produce DON [20,21]. Therefore, H_2_O_2_ has been recognized as an important factor in increasing the accumulation of DON. However, it is worth noting that H_2_O_2_ is not only a conventional signal compound in *F. graminearum,* but also a key signal molecule in plants, especially under environmental pressure [22,23]. Nevertheless, the indirect effect of these fungicides on DON production after stressing wheat to produce H_2_O_2_ is still unclear, even though this scientific question was raised as early as 2010 [19,24,25]. Furthermore, some pesticides, such as insecticides, are often mixed or rotated with fungicides during FHB epidemic or bioaccumulated by wheat spikes [26,27], and these nontarget pesticides may also cause the accumulation of physiological H_2_O_2_ in *F. graminearum* or in wheat, which is likely to increase the risk of DON accumulation. However, the actual impact is largely unknown. Exploring these problems will help to explain whether these factors are one of the reasons for the increase in DON levels in the field.

Therefore, we selected five pesticides, epoxiconazole, hexaconazole, imidacloprid, isoproturon, and mesosulfuron-methyl, and conducted in vitro and in vivo induction experiments, wheat oxidative stress experiments, greenhouse experiments, and wheat bioaccumulation experiments to explore: (1) the indirect effect of fungicides on DON production after stressing wheat to produce H_2_O_2_; (2) the direct and indirect effects of nontarget pesticides on DON accumulation; and (3) the risk assessment of DON according to the wheat bioaccumulation of residual pesticides. In particular, the herbicides were selected with the aim to enlarge the experimental effect. We hope that the obtained results will help broaden the understanding of the indirect effect of fungicides on DON levels and the effect of nontarget pesticides on DON accumulation and contribute to the scientific application of pesticides to reduce DON levels and food contamination risks.

## 2. Materials and Methods

### 2.1. Chemicals, Fungal Strains, and Culture Media

Epoxiconazole (99.1%), hexaconazole (99.0%), imidacloprid (99.0%), isoproturon (99.0%), and mesosulfuron-methyl (98.0%) were obtained from Beijing Qinchengyixin Technology Development Co., Ltd., Beijing, China. HPLC-grade acetonitrile was purchased from Tianjin Shield Fine Chemical Co., Ltd., Tianjin, China. All technical-grade pesticides were dissolved in HPLC-grade acetonitrile at 20,000 mg L^−1^ or 2000 mg L^−1^ and stored at 4 °C before further use. Epoxiconazole formulation (25%, suspension concentrate, SC) and imidacloprid formulation (5%, emulsifiable concentrate, EC) were purchased from Qingdao Zhongda Agricultural Technology Co., Ltd., Qingdao, China and Hailir Pesticides and Chemicals Group Co., Ltd., Qingdao, China, respectively. The wild-type strain of *F. graminearum* (PH-1, NCBI:txid229533) preserved in our laboratory was used in this study and cultured on potato dextrose agar (PDA) at 25 °C. Carboxymethyl cellulose media (CMC) was used to prepare conidial suspensions of PH-1 [28]. Trichothecene biosynthesis-inducing media (TBI) were used for in vitro induction experiments [28]. The remaining reagents were all analytical grade with a purity >98%.

### 2.2. Pesticide Sensitivity Tests

A series of concentrations of pesticides were added to the melted PDA media to reach the concentrations in Table S1. For nontarget pesticides, the maximum concentrations were the recommended field application rates (FRs). Mycelial plugs (4 mm in diameter) taken from the margins of actively growing colonies (3 days old) were transferred to the center of the solidified PDA plates. The media with acetonitrile was regarded as the control, and all the treatments were repeated with three replicates and cultured in darkness at 25 °C in an incubator (SPX-280, Ningbo Jiangnan Instrument Factory, Ningbo, China). The mean colony diameter was measured after 3 days of culture, and growth inhibition (%) was calculated as follows:

Inhibition (%) = (colony diameter in the control − colony diameter in the treatment) × 100/(colony diameter in the control − 4 mm).(1)

The EC_10_, EC_50_, and EC_90_ values were calculated based on the linear regression of growth inhibition (%) on log-transformed pesticide concentrations [1].

### 2.3. In Vitro Induction Experiments

Conidial suspensions of PH-1 were transferred into 250 mL flasks containing 100 mL TBI media (conidial density: 1 × 10^6^/mL). Then, flasks were incubated at 28 °C in darkness in a rotary shaker (150 rpm). After the mycelia were initially grown, pesticides were added into the cultures to reach the concentrations shown in Table S2, which were based on the EC_10_, EC_50_, and EC_90_ values or the FR. Treatment without pesticides was regarded as a control, and all the treatments were repeated twice with three replicates. After incubation for an additional 5 days, the mycelia were collected, dried, weighed, and stored at −80 °C upon extraction. The cultures were collected to detect H_2_O_2_ and DON. The production of H_2_O_2_ or DON was expressed as a ratio of H_2_O_2_ or DON content to mycelia dry weight (mg kg^−1^ DW).

### 2.4. Relative Expression of Tri5

For treatments with significantly increased DON production, total RNA of mycelia was extracted using the SteadyPure Universal RNA Extraction Kit (Accurate Biology Co., Changsha, China). RNA (10 μg) was subjected to reverse transcription using a HiScript II First Strand cDNA Synthesis Kit (Vazyme Biotech, Nanjing, China). Tri5 expression was determined using qRT–PCR with the primers and real-time PCR procedure reported in our previous study (Table S3) [28]. The relative expression of the Tri5 gene was calculated according to the 2^−ΔΔCt^ method using actin as a reference gene (Table S3).

### 2.5. Validation of the Induction Effect In Vitro

Catalase was used to verify the in vitro stimulating effect of H_2_O_2_ induced by pesticides [20]. Based on the results of Section 2.3, pesticide treatments with significantly elevated DON and H_2_O_2_ production were selected for verification. The experimental procedure and pesticide concentrations were the same as those in Section 2.3 except that catalase was immediately added into the cultures to a final concentration of 1000 U/mL after adding pesticides, and catalase was added again on day 3 to eliminate H_2_O_2_. Treatment without pesticides and catalase was regarded as a control, and all the treatments were carried out in triplicate. The production of DON and the relative expression of the Tri5 gene were also determined.

### 2.6. Wheat Cultivation and Oxidative Stress Experiment

To shorten the experimental period, wheat seedlings were used in the oxidative stress experiment. Seeds of wheat (Jimai 22) were surface-sterilized with a 6% sodium hypochlorite solution for 10 min and rinsed thoroughly with deionized water. Then, the seeds were soaked in deionized water for 15 h and germinated in seedling trays for 7 days. Afterward, the seedlings were transferred to a ¼ Hogland nutrient solution to continue growth for another week in a growth chamber at a 28/25 °C day/night temperature and relative humidity of 80% at a light intensity of 250 μmol m^−2^ s^−1^ with a photoperiod of 16 h each day. Seedlings of similar size (root length 14 ± 1 cm; shoot height 20 ± 2 cm) were transplanted into 250 mL flasks (ten seedlings per flask) containing 150 mL of new nutrient solution containing pesticides. The application concentrations of pesticides are shown in Table S4, which is based on the environmental residual concentrations [29,30,31,32,33,34]. The flask mouth was plugged with absorbent cotton to fix the wheat seedlings until the wheat roots were immersed just below the surface of the solution, and then the flasks were wrapped with foil paper. The evaporated water was replenished every day. Treatments without pesticides were performed as a control. All treatments were carried out in triplicate and incubated in a growth chamber. The wheat seedlings were removed from the flasks at 1, 3, and 7 days, and the leaves were cut off. The content of H_2_O_2_ in leaves was determined immediately after sampling. The enzyme activities of superoxide dismutase (SOD), catalase (CAT), and peroxidase (POD) were measured following the method of Song et al. [35], Zhang et al. [36], and Wang et al. [37], respectively.

### 2.7. In Vivo Induction Experiments

The setting of nutrient solution, wheat seedlings, pesticide concentrations, and incubation conditions were the same as those in Section 2.6. Then, the wheat leaves were infected with 20 μL conidia suspension of PH-1 (1 × 10^6^/mL, containing 5 drops of Tween 20 per milliliter) after 1 day of pesticide uptake. The wheat seedlings were incubated for 6 days. The evaporated water was replenished every day. Treatments without pesticides were conducted as a control, and all treatments were carried out with eight replicates. Then, the leaves were cut off and ground into powder in a mortar with liquid nitrogen. The DON content was determined. DON production was expressed as a ratio of the absolute mass of DON to gene copy numbers of PH-1 (μg copies Fg^−1^). In addition, the accumulated concentrations of pesticides in wheat leaves were also determined for treatments with significantly increased DON production.

### 2.8. DNA Extraction and Population Abundance Quantification of PH-1

The genomic DNA of PH-1 mycelia on wheat leaves was extracted using the CTAB method [28]. The population abundance of PH-1 was quantified using qRT–PCR with the primers and real-time PCR procedure in Table S3 [38]. The specificity of the amplicons was verified with melting curve analyses. A standard curve was generated by using 10-fold serial dilutions of pure PH-1 DNA with known copy numbers. The amplification efficiencies of the target genes ranged from 97.59% to 110.70%, with all the *R*^2^ values greater than 0.9934. The target gene copy numbers of the samples were calculated from the standard curves.

### 2.9. Greenhouse Validation Experiment

Greenhouse cultivation (25 °C, humidity 70%) can eliminate the interference of environmental factors such as wind and rain, so it was used to confirm the induction effect of pesticides on DON levels. Field soil (0–15 cm) was collected from the experimental plots of Qingdao Agricultural University and sieved through a <2 mm sieve. Wheat was planted in the soil by pot planting (thinning to six seedlings per flowerpot). During the flowering stage of wheat, the spikes were artificially inoculated with the conidia of PH-1 (20 μL, 1 × 10^6^ conidia/mL) using the single flower injection method and then bagged for moisture [17]. After 3 days of infection, 25% epoxiconazole SC and 5% imidacloprid EC were diluted with water to the corresponding concentration in Table S4 and sprayed on the aboveground part of wheat. Treatments without pesticides were conducted as the control. Each treatment (10 pots of wheat per treatment) was repeated three times, and the wheat plants were irrigated every two days. Thirty of the diseased wheat ear samples (per treatment) with similar disease severity were collected after 10 days of pesticide application. To extract DON and DNA from wheat, the collected wheat ears were ground into powder in a mortar with liquid nitrogen. The genomic DNA of PH-1 mycelia on wheat ears was extracted using the CTAB method [28], and the population abundance of PH-1 was quantified as described in Section 2.8.

### 2.10. Bioaccumulation of Pesticides by Wheat Plants

One kilogram of field soil was weighed into the flowerpot, and epoxiconazole and imidacloprid were added separately to reach the concentrations shown in Table S4. The soil was stirred for 10 min to make the pesticide concentration uniform. Soil humidity was adjusted to 60% of the maximum water holding capacity. The wheats just heading were transplanted into flowerpots (six wheats per flowerpot) to grow to maturity in a greenhouse. Wheats were fixed with bamboo sticks and thread and replenished with water every two days. Treatments without pesticides were performed as controls, and each treatment had five repetitions. After harvest, the wheat ears were cut, crushed, and stored at −20 °C for further determination of pesticide residues.

### 2.11. Extraction and Detection of H_2_O_2_, DON, and Pesticides from Different Matrices

The concrete extraction and determination methods of H_2_O_2_, DON, and pesticides from different matrices are given in Supplementary Materials, Tables S5 and S6. The extraction efficiency of DON and pesticides was verified using recovery tests with two spiked levels set as in Table S7. Five replicates for each matrix per level were conducted. Satisfactory recoveries and relative standard deviations were obtained and are summarized in Table S7. The calibration curves showed good linearity for DON and pesticides in different matrices, with all *R^2^* values greater than 0.9956. The limit of detection and limit of quantitation were 0.004 and 0.01 mg kg^−1^, respectively, for both pesticides. The sample concentration was quantified via the external standard method with matrix-matched calibration curves.

### 2.12. Statistical Analysis

All data are expressed as the mean ± standard deviation. One-way analysis of variance followed by Duncan’s test were performed to compare the significant differences (*p* < 0.05) between different treatments and controls using SPSS statistical software package version 17.0 (IBM Corp., Armonk, NY, USA).

## 3. Results and Discussion

### 3.1. Pesticide Sensitivity Tests

The EC_10_, EC_50_, and EC_90_ values were used as indices to evaluate the sensitivity of *F. graminearum* to pesticides. The calculated EC_10_, EC_50_, and EC_90_ values of epoxiconazole were 0.274 ± 0.013, 0.723 ± 0.008, and 3.610 ± 0.129 mg L^−1^, respectively (Table 1). The values of hexaconazole were 0.184 ± 0.013, 1.170 ± 0.079, and 7.490 ± 0.515 mg L^−1^, respectively (Table 1). Although the maximum test concentrations of imidacloprid, isoproturon, and mesosulfuron-methyl reached the recommended field application rates, they did not show any growth-inhibiting effect on *F. graminearum*.

### 3.2. Accumulation of H_2_O_2_ and DON In Vitro and Expression of the Tri5 Gene after Pesticide Application

**Figure 1:**
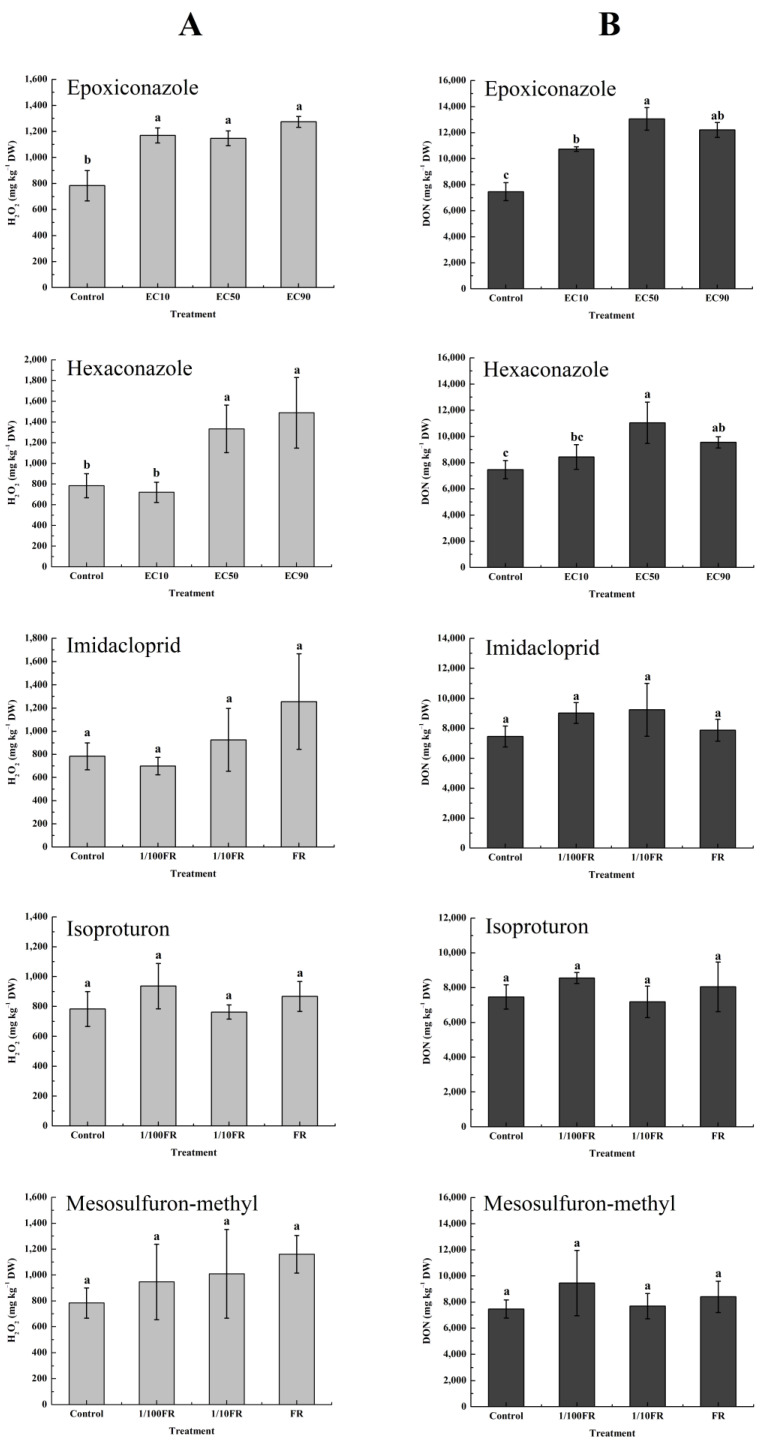
The release of H_2_O_2_ from *F. graminearum* after being treated with five pesticides (mg kg^−1^ DW) (**A**) and the production of DON in vitro after being treated with five pesticides (mg kg^−1^ DW) (**B**). Values are shown as mean ± SD, *n* = 6. The letters indicate the differences between the treatment and control (*p* < 0.05, Duncan’s test).

An in vitro induction experiment was conducted to study the accumulation of H_2_O_2_ and DON and the expression of the Tri5 gene after pesticide application. The release of H_2_O_2_ from *F. graminearum* under pesticide stress is shown in Figure 1A. The application of epoxiconazole significantly induced an increase in H_2_O_2_ by 46–63% at three sublethal concentrations. However, there was no significant difference between epoxiconazole treatments. For hexaconazole, two concentrations, EC_50_ and EC_90_, stimulated the accumulation of H_2_O_2_ (70–90%). The nontarget pesticides imidacloprid, isoproturon, and mesosulfuron-methyl did not stimulate H_2_O_2_ release from *F. graminearum* at different concentrations.

Figure 1B shows the accumulation of DON under pesticide stress. The application of epoxiconazole greatly increased the production of DON (44–75%) under the three concentrations. In contrast, hexaconazole did not stress the production of DON at low concentrations but significantly triggered the accumulation of DON at the concentrations of EC_50_ and EC_90_ (28–48%), which was consistent with the results of H_2_O_2_. For nontarget pesticides, imidacloprid, isoproturon, and mesosulfuron-methyl did not influence DON accumulation at the different concentrations.

**Figure 2:**
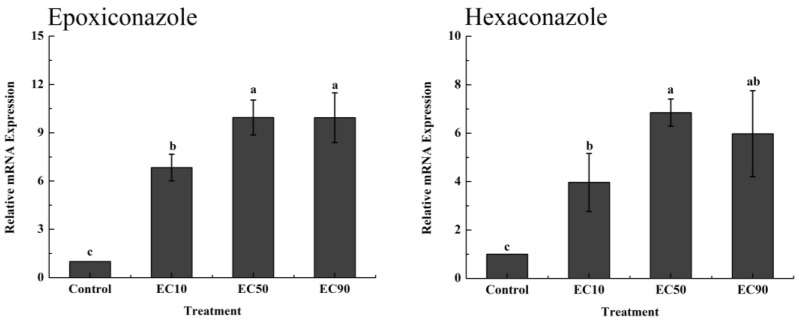
Relative expression of the Tri5 gene in vitro after treatment with epoxiconazole and hexaconazole. Values are shown as mean ± SD, *n* = 6. The letters indicate the differences between the treatment and control (*p* < 0.05, Duncan’s test).

qRT–PCR was performed to further confirm the in vitro induction effect of epoxiconazole and hexaconazole on DON accumulation. The relative expression of the Tri5 gene related to DON synthesis was determined because the Tri5 gene encodes a sesquiterpene cyclase that controls the first step of DON biosynthesis [2,39]. As Figure 2 shows, compared with the control, Tri5 expression was upregulated by 6.8-fold, 9.9-fold, and 9.9-fold under epoxiconazole treatment at EC_10_, EC_50_, and EC_90_, respectively. For treatment with hexaconazole, different from the measurement results of DON, the expression of the Tri5 gene was not only upregulated at the concentrations of EC_50_ and EC_90_, but also upregulated at the concentration of EC_10_. The expression levels of Tri5 were upregulated at EC_10_, EC_50_, and EC_90_ by 3.9-fold, 5.8-fold, and 6.9-fold, respectively.

Similar to our results, Duan et al. also observed that epoxiconazole induced the production of H_2_O_2_ and DON in vitro at EC_50_ and EC_90_ concentrations [17]. Other triazole fungicides, tebuconazole, prothioconazole, and flutriafol, have also been shown to induce an increase in DON production and Tri5 gene expression in *F. graminearum* in vitro [19,24,40]. The above results show that epoxiconazole and hexaconazole exerted selective pressure on *F. graminearum*. The inhibition of ergosterol biosynthesis by epoxiconazole and hexaconazole may result in an increased cell permeability, which in turn leads to an increased H_2_O_2_ release in the TBI medium, thus activating the mechanism of DON biosynthesis [19]. However, other nontarget pesticides have no direct induction effect on the accumulation of DON in vitro.

### 3.3. Validation of the Induction Effect of H_2_O_2_ Induced by Epoxiconazole and Hexaconazole In Vitro

**Figure 3:**
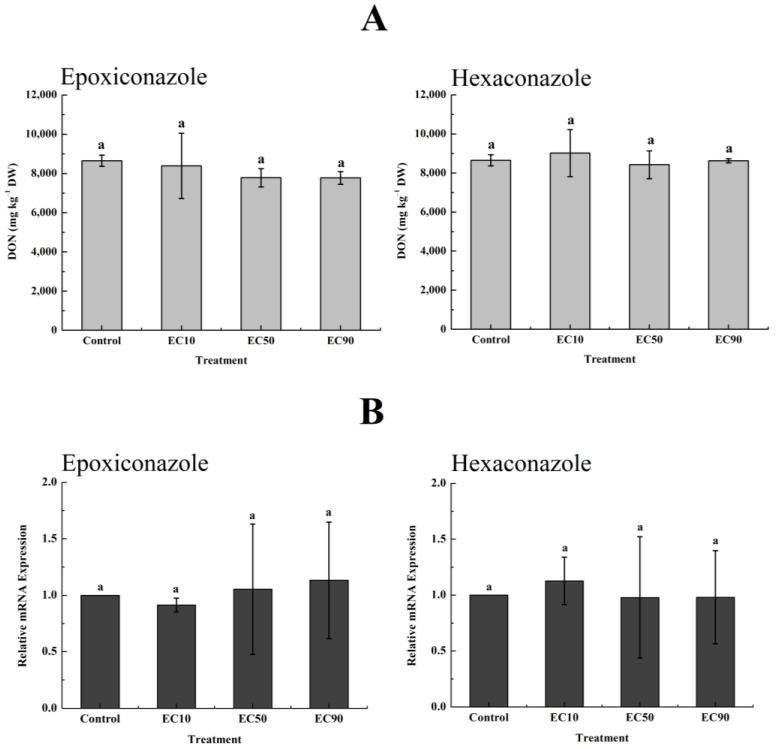
The production of DON in vitro after treatment with epoxiconazole and hexaconazole under the action of catalase (mg kg^−1^ DW) (**A**) and the relative expression of the Tri5 gene in vitro after treatment with epoxiconazole and hexaconazole under the action of catalase (**B**). Values are shown as mean ± SD, *n* = 3. The letters indicate the differences between the treatment and control (*p* < 0.05, Duncan’s test).

To further confirm the stimulatory effect of H_2_O_2_ induced by epoxiconazole and hexaconazole on DON production, we carried out a verification experiment using catalase. Figure 3A shows that the production of DON did not increase significantly under the action of catalase whether it was treated with epoxiconazole or hexaconazole. However, the production of DON also did not decrease compared to the control. The relative expression of Tri5 also did not change significantly compared to the control (Figure 3B). The above results prove that H_2_O_2_ stressed by epoxiconazole and hexaconazole had a stimulating effect on the production of DON in vitro. However, DON production was still affected by epoxiconazole and hexaconazole in the presence of catalase; otherwise, DON production and Tri5 expression would be lower than that of the control, similar to the results of Ponts et al. [20]. Similarly, Audenaert et al. observed that the production of DON for prothioconazole treatment was significantly reduced by catalase but did not show any difference compared to the control [19]. Since ergosterol and DON have a common precursor, farnesyl pyrophosphate, more farnesyl pyrophosphate will accumulate and flow to the DON synthesis pathway when ergosterol synthesis is inhibited by triazole fungicides [41]. This may be the reason why the accumulation of DON was still not lower than that in the control after catalase application. The above results demonstrate that in addition to the effects of the fungicides themselves, epoxiconazole- and hexaconazole-induced H_2_O_2_ can stimulate DON production in vitro.

### 3.4. Oxidative Stress of Pesticides on Wheat Plants

**Figure 4:**
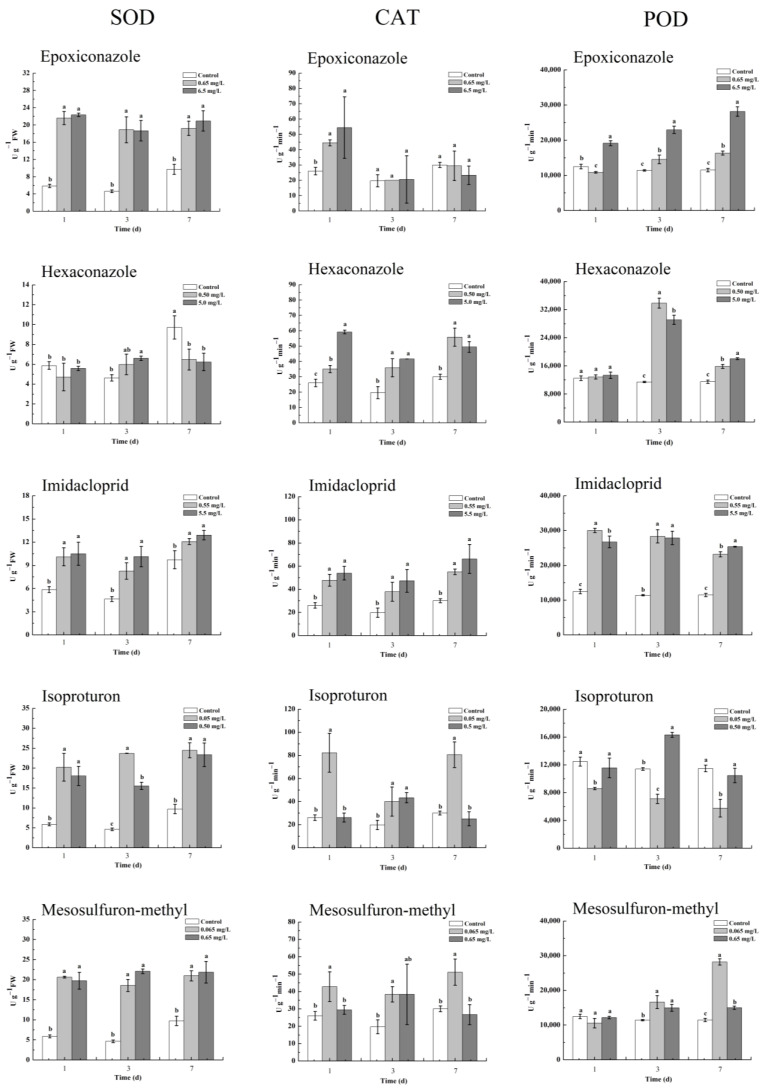
The activities of the antioxidant enzymes SOD, CAT, and POD in wheat leaves under different levels of pesticide oxidative stress. Values are shown as mean ± SD, *n* = 3. The letters indicate the differences between the treatment and control (*p* < 0.05, Duncan’s test).

Plants exposed to various pesticide stresses are forced to generate high levels of reactive oxygen species such as O_2_^−^ and H_2_O_2_ and activate a series of antioxidant enzymes [42]. SOD is the first and major enzyme that catalyzes the dismutation of O_2_^−^ to H_2_O_2_ and O_2_ [23]. The produced H_2_O_2_ can be converted into H_2_O and O_2_ by a variety of H_2_O_2_ scavenging enzymes, such as CAT and POD [23]. In this study, the oxidative stress on wheat plants caused by pesticides at one and ten times the maximum environmental residual concentration was determined. Figure 4 shows that epoxiconazole significantly increased the SOD activity by 0.97 to 3.1 times compared with the control under the two concentrations from the first day to the seventh day. In contrast, CAT activity increased only on the first day under epoxiconazole treatment, and the activity of POD increased significantly (0.27- to 1.45-fold) during the whole experiment and increased more at high concentrations than at low concentrations. For hexaconazole, the activity of SOD basically did not increase under the two concentrations, while the activities of CAT and POD increased significantly to varying degrees. Similarly, imidacloprid significantly increased the activities of SOD, CAT, and POD at both concentrations. Isoproturon also increased the activity of SOD, but the activity of CAT significantly increased only at low concentrations, and the activity of POD ultimately did not increase. For mesosulfuron-methyl, the activity of SOD increased at both concentrations, but CAT activity increased only at low concentrations, while POD activity increased at both concentrations on days 3 and 7.

**Figure 5:**
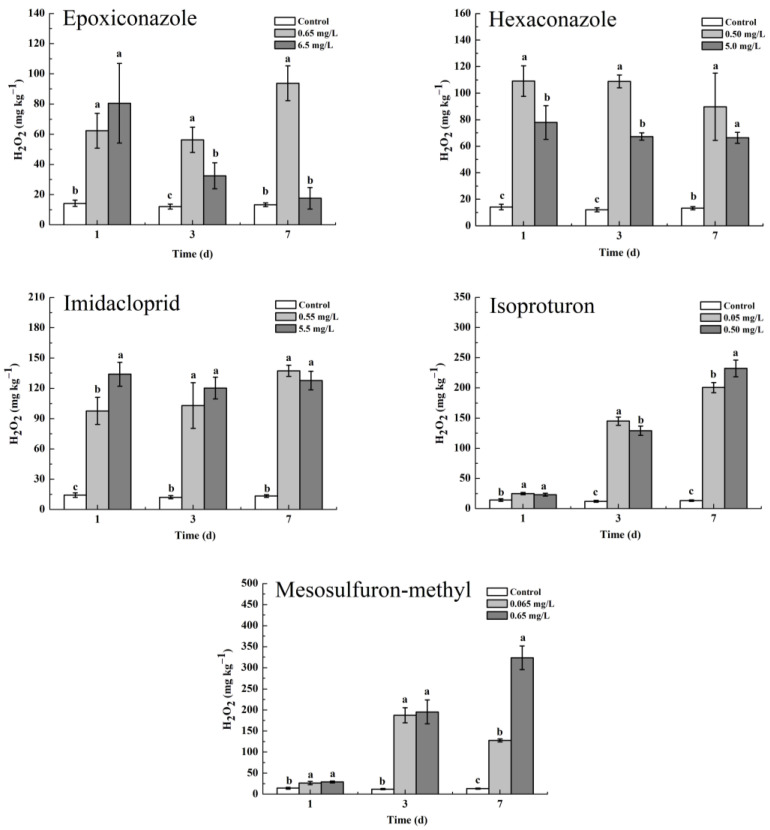
The accumulation of H_2_O_2_ in wheat leaves under the oxidative stress of five pesticides (mg kg^−1^). Values are shown as mean ± SD, *n* = 3. The letters indicate the differences between the treatment and control (*p* < 0.05, Duncan’s test).

The above results reveal that O_2_^−^ was produced in wheat plants under the stress of epoxiconazole, imidacloprid, isoproturon, and mesosulfuron-methyl, which further activated the activity of SOD, and O_2_^−^ was gradually decomposed into H_2_O_2_ [23]. In turn, the activities of CAT and POD were further activated by H_2_O_2_ to varying degrees, which also indicated that the generated H_2_O_2_ was decomposed at the same time [42]. Although the activity of SOD did not increase after treatment with hexaconazole, the activities of CAT and POD were also activated, reflecting the actual presence of excess H_2_O_2_ in wheat leaves. Under the action of different antioxidant enzymes, the accumulation of H_2_O_2_ in wheat leaves is shown in Figure 5. Each pesticide significantly increased the accumulation of H_2_O_2_ in wheat leaves at different concentrations. The herbicides isoproturon and mesosulfuron-methyl stressed the accumulation of H_2_O_2_ more obviously than the other three pesticides on the third and seventh days.

Similar to our results, Wu et al. and Honorato Júnior et al. observed that the application of epoxiconazole could induce the increasing activities of SOD, CAT, and POD as well as H_2_O_2_ content in wheat and coffee leaves [43,44]. Dubey et al. found that hexaconazole increased the activities of antioxidant enzymes and the level of H_2_O_2_ in barley [45]. Sharma et al. and Shakir et al. found that imidacloprid could increase the activities of SOD, CAT, POD, and H_2_O_2_ levels in the shoots of *Brassica juncea* L. plants and tomato seedlings [46,47]. Yin et al. reported that isoproturon induced a general increase in the activities of SOD, POD, and CAT in wheat plants at low concentrations [48]. The present results indicated that the tested pesticides, especially herbicides, caused oxidative stress on wheat seedlings and stressed wheats to accumulate excessive physiological H_2_O_2_, which is likely to further trigger DON production when wheats are infected by *F. graminearum*.

### 3.5. Effects of the Pesticide-Induced Oxidative Stress Response on DON Accumulation In Vivo

**Figure 6:**
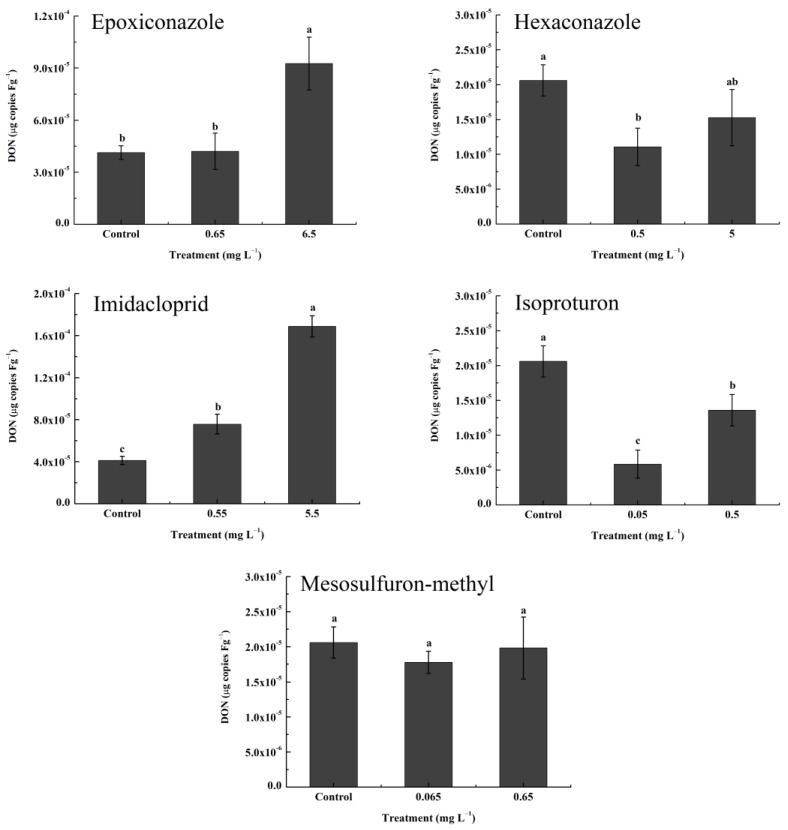
The production of DON in vivo under treatment with epoxiconazole, hexaconazole, imidacloprid, isoproturon, and mesosulfuron-methyl (μg copies Fg^−1^). Values are shown as mean ± SD, *n* = 8. The letters indicate the differences between the treatment and control (*p* < 0.05, Duncan’s test). (note: The experiment was conducted in two batches. Epoxiconazole and imidacloprid were the first batch, and hexaconazole, isoproturon and mesosulfuron-methyl were the second batch, with independent controls).

An in vivo induction experiment was conducted to further study the effect of pesticide-induced H_2_O_2_ in wheat on the production of DON. To avoid direct contact between the pesticides and PH-1 at the beginning and to ensure sufficient oxidation pressure during the experiment, we adopted the root application method for the in vivo experiment. The result of in vivo induction is shown in Figure 6. Compared with the control, DON production on wheat leaves did not increase after treatment with a low concentration of epoxiconazole but increased by 1.3 times at a high concentration of epoxiconazole. Conversely, when treated with hexaconazole, DON levels showed a downward trend but decreased significantly (nearly 50%) only at low concentrations. Interestingly, imidacloprid, as a nontarget pesticide, significantly increased the DON levels (1.8- to 4.1-fold) at both concentrations, which contrasted with isoproturon, which significantly reduced DON levels (nearly 50%) at both concentrations. For mesosulfuron-methyl, the level of DON was not affected by its application in wheat.

In contrast to our expectation, although H_2_O_2_ already existed in wheat leaves when PH-1 started producing DON according to the wheat oxidative stress experiment, the corresponding DON level did not increase significantly in some pesticide treatments. For epoxiconazole, the accumulation of H_2_O_2_ in wheat leaves was the highest after 3 days of treatment at low concentrations, but the corresponding DON level did not significantly increase but increased clearly at high concentrations. For hexaconazole, although the concentration of H_2_O_2_ in wheat highly increased, the level of DON in wheat decreased significantly, which was also different from the in vitro experiments. These results indicate that the excess H_2_O_2_ in wheat leaves stressed by the two fungicides did not play a role in stimulating DON production. The increase in DON at high concentrations of epoxiconazole was most likely caused by the stimulation of epoxiconazole itself because its accumulation in wheat leaves was close to the EC_10_ (Table 1 and Table 2), while the results for hexaconazole suggest that there may be other factors affecting the production of DON.

For nontarget pesticides, the accumulation of H_2_O_2_ in wheat leaves was the highest after treatment with isoproturon and mesosulfuron-methyl, while the level of DON in these treatments did not increase, which also indicated that the accumulated H_2_O_2_ in wheat did not stimulate the production of DON. Unexpectedly, the level of DON in wheat leaves increased substantially after imidacloprid treatment, under the premise that imidacloprid had no direct effect on PH-1. However, based on the above analysis, it is prudent to say that it could not be attributed to the accumulation of H_2_O_2_ in wheat. Due to the fact that H_2_O_2_ may be an important signaling molecule required for the functional adaptation mechanism of wheat, it has a complex physiological role, such as causing changes in secondary metabolism in wheat [49]. Therefore, under the stimulation of different pesticides, the accumulation of DON in wheat may be influenced by a combination of many complex factors within the wheat itself, not just hydrogen peroxide itself. It has been reported that secondary metabolites of wheat, such as sucrose and polyamines, can enhance the biosynthesis of DON [50], while benzoxazinoids, flavonoids, salicylic acid, jasmonic acid, proline, and alanine can reduce the accumulation of DON [51,52]. The unexpected result of imidacloprid or other tested pesticides may be due to its influence on the secondary metabolism of wheat, thus indirectly affecting the accumulation of DON in wheat.

Similarly, there are some other reports which state that nontarget pesticides affect the production of mycotoxins. Scarpino et al. recorded that the application of alpha-cypermethrin and a mixture of lambda-cyhalothrin and chlorantraniliprole led to an increase in DON and zearalenone contamination in corn fields [53]. Felix D’Mello et al. found that dichlorvos, landrin, malathion, and diazinon could inhibit the production of aflatoxin B_1_ by Aspergillus parasiticus, while naled could effectively reduce zearalenone levels in pure cultures of *F. graminearum* or on maize kernels [54]. Ponts et al. reported that paraquat exerted a strong inhibitory effect, as DON and 3/15-ADON accumulated nearly 10-fold less than in the controls in liquid cultures of *F. graminearum* [21]. Abbas et al. reported a potential plant-based insecticide derived from *Zanthoxylum bungeanum*, which is composed of four flavonoids and can decrease *F. graminearum* growth and abrogate DON production [55].

### 3.6. Greenhouse Validation Experiment

**Figure 7:**
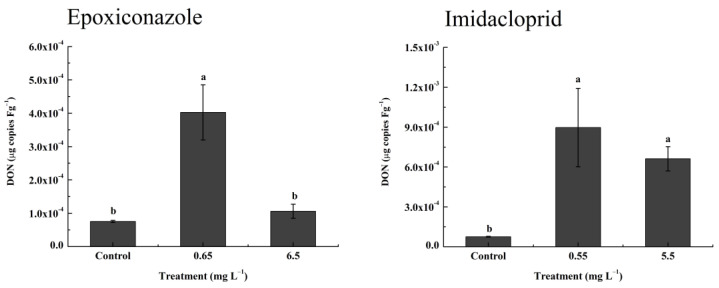
The production of DON under epoxiconazole and imidacloprid treatment in the greenhouse experiment (μg copies Fg^−1^). Values are shown as mean ± SD, *n* = 3. The letters indicate the differences between the treatment and control (*p* < 0.05, Duncan’s test).

A greenhouse experiment was used to confirm the induction effect of epoxiconazole and imidacloprid on DON levels in vivo. As Figure 7 shows, after treatment with epoxiconazole, DON levels in wheat ears increased only at the low concentration (5.3-fold), while a high concentration of epoxiconazole did not stimulate the production of DON, which was different from the results of the in vivo induction experiment. Different actual exposure concentrations caused by different application methods led to different results. The low exposure concentration of epoxiconazole in the greenhouse experiment was close to the actual accumulation concentration of the high concentration of epoxiconazole in the in vivo induction experiment (Table 2), while the high exposure concentration of epoxiconazole in the greenhouse experiment exceeded the EC_90_ (Table 1) and may inhibit the production of DON. Imidacloprid exhibited significant stimulating effects on DON production (8.7- to 11.9-fold) in wheat ears at the two concentrations. The above results confirm the stimulatory effect of the sublethal concentration of epoxiconazole on DON levels and the indirect stimulatory effect of the nontarget pesticide imidacloprid on DON accumulation. Considering that the application time of imidacloprid often overlaps with the occurrence time of FHB, it greatly increases the risk of DON contamination.

### 3.7. Risk Assessment of DON Based on Bioaccumulation of Pesticides by Wheat Plants

The bioaccumulation of epoxiconazole and imidacloprid by wheat ears under one and ten times the environmental maximum residual concentrations was simulated to estimate the potential effective range of accumulation. As Table 3 shows, the bioaccumulation concentration of epoxiconazole in wheat ears ranged from 25.10 μg kg^−1^ to 36.80 μg kg^−1^, while the concentration range of imidacloprid was 9.45–957.00 μg kg^−1^.

Comparing Table 2 and Table 3, the concentration of epoxiconazole in wheat ears was much lower than its accumulation in wheat leaves at an application concentration of 6.50 mg L^−1^ and even lower than its accumulation in wheat leaves at an application concentration of 0.650 mg L^−1^, which means that the epoxiconazole in wheat ears did not reach the dose that caused an increase in DON. For imidacloprid, although its concentration in wheat ears was up to 0.957 mg kg^−1^, it was much lower than its accumulated concentrations in leaves at the corresponding concentration. However, the risk remains because the in vivo stimulation effect of imidacloprid was indirect, which means that the low concentration of imidacloprid may still stimulate the increase in DON. More importantly, the above analysis was based on root uptake at residual environmental concentrations. In fact, the application methods of epoxiconazole and imidacloprid in the field are mainly stem and leaf spray, which greatly enhance the risk of DON rise in the field.

## 4. Conclusions

This study mainly explored the direct and indirect effects of fungicides and nontarget pesticides on DON accumulation. Epoxiconazole and hexaconazole could directly stimulate the production of H_2_O_2_ by *F. graminearum*, which can further stimulate the accumulation of DON in vitro. However, these two fungicides did not have an indirect effect on DON production after stressing wheat to produce H_2_O_2_. The nontarget pesticides imidacloprid, isoproturon, and mesosulfuron-methyl had no direct induction effect in vitro, and the excessive H_2_O_2_ in wheat under these pesticide stresses did not indirectly stimulate an increase in DON. Therefore, pesticide-induced H_2_O_2_ in vitro is an important factor in stimulating DON production by *F. graminearum*, but the environmentally induced H_2_O_2_ level in wheat is not sufficient to stimulate DON production. The application of pesticides in the field is mainly prevention, so according to our results, the increase in H_2_O_2_ in wheat caused by an early application of pesticides will not cause the accumulation of DON. However, some nontarget pesticides, such as imidacloprid, may also affect the production of DON by causing changes in the secondary metabolism of wheat. From the perspective of food safety, the reduction in DON pollution levels in wheat is what we expect. Therefore, the impact of nontarget pesticides on DON accumulation still needs more attention. Because the DON level in the field environment is also affected by many environmental factors, such as humidity, temperature, and rainfall, whether the present results can represent the real situation in the field remains to be studied.

## Figures and Tables

**Table 1:** The EC_10_, EC_50_, and EC_90_ values of epoxiconazole and hexaconazole. Values are shown as mean ± SD, *n* = 3.

	Sublethal Concentrations	Value (mg L^−1^)
	EC_10_	0.274 ± 0.013
Epoxiconazole	EC_50_	0.723 ± 0.008
	EC_90_	3.610 ± 0.129
	EC_10_	0.184 ± 0.013
Hexaconazole	EC_50_	1.170 ± 0.079
	EC_90_	7.490 ± 0.515

**Table 2:** The accumulation concentrations of epoxiconazole and imidacloprid in wheat leaves in in vivo induction experiments. Values are shown as mean ± SD, *n* = 8.

Pesticides	Application Concentrations (mg L^−1^)	Accumulation Concentrations (mg kg^−1^)
Epoxiconazole	0.650	0.080 ± 0.004
	6.500	0.331 ± 0.015
Imidacloprid	0.550	2.070 ± 0.374
	5.500	14.300 ± 0.270

**Table 3:** The bioaccumulation concentrations of epoxiconazole and imidacloprid in wheat ears. Values are shown as mean ± SD, *n* = 5.

Pesticides	Application Concentrations (mg kg^−1^)	Accumulation Concentrations (μg kg^−1^)
Epoxiconazole	0.65	25.10 ± 3.60
	6.50	36.80 ± 1.93
Imidacloprid	0.55	9.45 ± 1.80
	5.50	957.00 ± 67.90

## Data Availability

Data are contained within the article or Supplementary Materials.

## Supplementary Materials

The following supporting information can be downloaded at: https://www.mdpi.com/article/10.3390/toxics11090768/s1, Table S1: Concentration ranges in pesticide sensitivity tests; Table S2: The concentrations of pesticides in in vitro induction experiments; Table S3: Primers and real-time PCR procedure used in this study; Table S4: Environmental residual concentrations of pesticides and one and ten times the pesticide application concentrations; Table S5: The standard curve reaction system of the detection method of H_2_O_2_; Table S6: UPLC–MS/MS conditions of the analytes studied; Table S7: Recoveries and RSD values for DON, epoxiconazole, and imidacloprid in two spiked levels from different matrices; Supplementary Materials File S1: The extraction and detection methods of H_2_O_2_, DON, and pesticides from different matrixes.
