# Supplementary material for: Effects of Fungicides and Nontarget Pesticides on Accumulation of the Mycotoxin Deoxynivlenol in Wheat
Source: Toxics. 2023 Sep 10;11(9):768. doi: 10.3390/toxics11090768 (PMC10535342; doi:10.3390/toxics11090768)
Supplement: Supplementary file 1 [file toxics-11-00768-s001.zip › Supplementary Material 1.pdf]

Supplementary Material 1

The direct and indirect effects of fungicides and nontarget pesticides on deoxynivalenol accumulation

Chao Ju[1], Fan Jiang[1], Yuan Gao[1], Tongwu Chen[1], Jiakuo Cao[1], Junbo Lv[1], Yanxiang Zhao[1], Yongquan Zheng[1,*], Wei Guo[2,*] and Jinguang Huang[1,*]

1. Shandong Engineering Research Center for Environment-Friendly Agricultural Pest Management, College of Plant Health and Medicine, Qingdao Agricultural University, Qingdao 266109, China;
2. Institute of Food Science and Technology, Chinese Academy of Agricultural Sciences/Key Laboratory of Agro-products Quality and Safety Control in Storage and Transport Process, Ministry of Agriculture and Rural Affairs, Beijing, 100193, China;

* Correspondence: zhengyongquan@qau.edu.cn; Tel.: +86-532-58957380;

guowei01@caas.cn; Tel.: +86-010-62815925;

jghuang@qau.edu.cn; Tel.: +86-532-58957365;

1. The extraction and detection method of H_2O_2

(1) Preparation of standard curve

Reaction solutions including 10 mM phosphate buffer (PH 7.0), 1 M KI, 10 mM (NH)MoO were prepared in advance. Then take seven 5-mL centrifuge tubes, add the reaction solutions according to the **Table S5**, shake gently, and let stand for 15 minutes. The absorbance value of the final reaction solution was measured at a wavelength of 352 nm, and a standard curve was made.

(2) Extraction and determination of H_2O_2 from the TBI medium

The liquid medium was collected and centrifuged at 4000 rpm for 5 min. Then, 1.5 mL of the upper layer was filtered through 0.45-μm nylon syringe filters and transferred 0.5 mL filtrate to a 5-mL centrifuge tube. Then add 0.5 mL of 10 mM H_3PO_4, 1 mL of 1 M KI, 0.1 mL of 10 mM (NH)MoO, shake gently, and let stand for 15 minutes. The absorbance value of the final reaction solution was measured at a wavelength of 352 nm. All the sample concentration was quantified by the standard curve.

(3) Extraction and determination of H_2O_2 from wheat leaves

Two grams of wheat leaves were cut off and ground into powder in a mortar with liquid nitrogen. Transferred the powder into a 50-mL centrifuge tube. Then add 10 mL of precooled 5% TCA solution, mix well, and centrifuged at 12000 rpm for 15 minutes. Then, 5 mL of the upper layer was transferred to a 10-mL centrifuge tube and purified with 200 mg of GCB to reduce the interference of pigments. The resulting mixture centrifuged at 4000 rpm for 1 min, and the resulting supernatant was

diluted 5 times with phosphate buffer. Then transferred 0.5 mL diluted solution to a 5-mL centrifuge tube. Add 0.5 mL of 10 mM H_3PO_4, 1 mL of 1 M KI, 0.1 mL of 10 mM (NH)MoO, shake gently, and let stand for 15 minutes. The absorbance value of the final reaction solution was measured at a wavelength of 352 nm. All the sample concentration was quantified by the standard curve.

2. The extraction and detection method of DON

(1) Extraction from TBI medium

The liquid medium was collected and centrifuged at 4000 rpm for 5 min. Then, 1.5 mL of the upper layer was transferred to a 2-mL centrifuge tube and purified with 15 mg of C_{18} and 5 mg of graphitized carbon black (GCB). The resulting mixture was vortexed and centrifuged again, and the resulting supernatant was filtered through 0.22-μm nylon syringe filters for UPLC–MS/MS analysis.

(2) Extraction from wheat leaves and ears

The leaves or ears of wheat were completely ground into powder in a mortar with liquid nitrogen, then homogenized in 8 mL of water for 2 min and then shaken vigorously for 10 min using a shaker. The mixture was centrifuged at 4000 rpm for 5 min. Then, 1.5 mL of the upper layer was transferred to a 2-mL centrifuge tube and purified with 25 mg of C_{18} and 10 mg of GCB. The resulting mixture was vortexed and centrifuged again, and the resulting supernatant was filtered through 0.22-μm nylon syringe filters for UPLC–MS/MS analysis. The absolute mass of DON was calculated from its concentration and the volume of water.

(3) **Detection method of DON**

All the sample extracts of DON were separated using a Waters Acquity UPLC system. This system included a Waters Acquity UPLC binary solvent manager, Acquity UPLC manager, and Acquity column heater equipped with a Waters Acquity UPLC BEH C_{18} column (2.1×100 mm, 1.7μm particle size; Milford, MA, USA). A gradient UPLC elution using methanol as mobile phase A and 0.1% formic acid aqueous solutions as mobile phase B was peformed. The analytes were separated at a flow rate of 0.4 mL min^{-1} with an elution gradient of 0.00 min/95% B, 2.00 min/95% B, 6.40 min/5% B, 8.40 min/5% B, 8.41 min/95% B, and 10.00 min/95% B. The column was held at 40℃ to decrease the viscosity, and the sample manager temperature was set to 5 °C. The injection volume was 1 μL. Separation and stabilization occurred within 10.00 min.

Concentration of the DON was determined by a triple-quadrupole mass spectrometer (TQD, Waters Crop., Milford, MA, USA) using the multiple reaction monitoring (MRM) mode and positive electrospray ionization (ESI+) mode. The following conditions were typical: the capillary voltage was set to 3.0 kV, and the source and desolvation temperatures were held at 120 and 350℃, respectively. The desolvation gas and cone gas (nitrogen, 99.95% purity) used flow rates of 600 and 50 L h^{-1}, respectively. The collision gas, which was high-purity argon, was maintained at 0.15 mL min^{-1} with a pressure of 2×10^{-3} mbar in the T-wave cell. The concrete MS-MS parameter for DON is listed in **Table S6**. The sample concentration was quantified by the external standard method with matrix-matched calibration curves.

The calibration curves showed good linearity for DON in TBI medium or in wheat leaves or ears with all R^2 values greater than 0.9956.

3. The extraction and detection method of pesticides

(1) Extraction of pesticides from wheat tissues

The wheat leaves powder (0.5 g) or ears powder (0.5 g) were weighed and homogenized in 2 mL of water and 8 mL of acetonitrile for 2 min and then shaken vigorously for 10 min using a shaker. Subsequently, 4 g of anhydrous $MgSO_4$ and 1 g of NaCl were added and vortexed for 1 min at maximum speed. The mixture was centrifuged at 4000 rpm for 5 min to acquire the supernatant. Then, 1.5 mL of the upper layer was transferred to a 2-mL centrifuge tube and purified with 25 mg of PSA, 25 mg of C_{18}, 150 mg of anhydrous $MgSO_4$ and 10 mg of GCB. The resulting mixture was vortexed and centrifuged again, and the resulting supernatant was filtered through 0.22-μm nylon syringe filters for UPLC–MS/MS analysis.

(2) Detection method of pesticides

All the sample extracts of pesticides were separated using a Waters Acquity UPLC system. This system included a Waters Acquity UPLC binary solvent manager, Acquity UPLC manager, and Acquity column heater equipped with a Waters Acquity UPLC BEH C_{18} column (2.1×100 mm, 1.7μm particle size; Milford, MA, USA). A gradient UPLC elution using methanol as mobile phase A and 0.1% formic acid aqueous solutions as mobile phase B was peformed. The analytes were separated at a flow rate of 0.4 mL min^{-1} with an elution gradient of 0.00 min/95% B, 2.00 min/95%

B, 6.40 min/5% B, 8.40 min/5% B, 8.41 min/95% B, and 10.00 min/95% B. The column was held at 40℃ to decrease the viscosity, and the sample manager temperature was set to 5 °C. The injection volume was 1 μL. Separation and stabilization occurred within 10.00 min.

Concentration of the pesticides was determined by a triple-quadrupole mass spectrometer (TQD, Waters Crop., Milford, MA, USA) using the multiple reaction monitoring (MRM) mode and positive electrospray ionization (ESI+) mode. The following conditions were typical: the capillary voltage was set to 3.0 kV, and the source and desolvation temperatures were held at 120 and 350℃, respectively. The desolvation gas and cone gas (nitrogen, 99.95% purity) used flow rates of 600 and 50 L h^{-1}, respectively. The collision gas, which was high-purity argon, was maintained at 0.15 mL min^{-1} with a pressure of 2×10^{-3} mbar in the T-wave cell. The concrete MS-MS parameter for these pesticides is listed in **Table S6**. The sample concentration was quantified by the external standard method with matrix-matched calibration curves. The calibration curves showed good linearity for pesticides in wheat leaves or wheat ears with all R^2 values greater than 0.9992.
