# Supplementary material for: Effects of Fungicides and Nontarget Pesticides on Accumulation of the Mycotoxin Deoxynivlenol in Wheat
Source: Toxics. 2023 Sep 10;11(9):768. doi: 10.3390/toxics11090768 (PMC10535342; doi:10.3390/toxics11090768)
Supplement: Supplementary file 1 [file toxics-11-00768-s001.zip › Supplementary Material-Tables.pdf]

Supplementary Material

The direct and indirect effects of fungicides and nontarget pesticides on deoxynivalenol accumulation

Chao Ju[1], Fan Jiang[1], Yuan Gao[1], Tongwu Chen[1], Jiakuo Cao[1], Junbo Lv[1], Yanxiang Zhao[1], Yongquan Zheng[1,*], Wei Guo[2,*] and Jinguang Huang[1,*]

a. Shandong Engineering Research Center for Environment-Friendly Agricultural Pest Management, College of Plant Health and Medicine, Qingdao Agricultural University, Qingdao 266109, China;

b. Institute of Food Science and Technology, Chinese Academy of Agricultural Sciences/Key Laboratory of Agro-products Quality and Safety Control in Storage and Transport Process, Ministry of Agriculture and Rural Affairs, Beijing, 100193, China;

* Correspondence: zhengyongquan@qau.edu.cn; Tel.: +86-532-58957380;

guowei01@caas.cn; Tel.: +86-010-62815925;

jghuang@qau.edu.cn; Tel.: +86-532-58957365;

Table S1. Concentration ranges in pesticide sensitivity tests.

Pesticides	Concentration (mg kg^{-1})
Epoxiconazole	0.1, 0.2, 0.4, 0.8, 1.6, 3.2, 6.4
Hexaconazole	0.1, 0.2, 0.4, 0.8, 1.6, 3.2, 6.4
Imidacloprid	0.75, 1.5, 3.0, 6.0, 12.0, 24.0, 120.0
Isoproturon	0.8, 1.6, 3.2, 6.4, 16.0, 32.0, 160.0
Mesosulfuron-methyl	1.2, 2.4, 4.8, 12.0, 24.0, 48.0, 240.0

Table S2. The concentrations of pesticides in *in vitro* induction experiments.

Pesticides	Concentration (mg kg^{-1})		
Epoxiconazole	0.3	0.8	3.6
Hexaconazole	0.2	1.2	7.5
Imidacloprid	1.2	12.0	120
Isoproturon	1.6	16.0	160
Mesosulfuron-methyl	2.4	24.0	240

Table S3. Primers and real-time PCR procedure used in this study.

Application	Primer name	Sequence (5'→3')	Mixed reaction liquid	Real-time PCR procedure	Product size
Internal control	actin-RT-F	TAGCATCGCATCTCATCAC	10 µL of 2 × SYBR Green PCR Master Mix, 0.4 µL of each forward and reverse primer (10 µM), 1 µL of 40-fold diluted template cDNA, 8.2 µL of RNase-free ddH_2O	3 s at 95 °C, followed by 40 cycles of 10 s at 95 °C, 30 s at 60°C. Data collection at 60 °C	150 bp
	actin-RT-R	CAGCAACTTCTTCCTCCAT			
Tri5 expression	Tir5-RT-F	GATGGAGAACTGGATGGT	10 µL of 2 × SYBR Green PCR Master Mix, 0.4 µL of each forward and reverse primer (10 µM), 1 µL of 40-fold diluted template DNA, 8.2 µL of RNase-free ddH_2O	3 s at 95 °C, followed by 40 cycles of 10 s at 95 °C, 30 s at 60°C. Data collection at 60 °C	168 bp
	Tri5-RT-R	TGCTTAGACGAGTGTAGG			
Population abundance of PH-1	Fg16N-F	ACAGATGACAAGATTCAGGCACA	10 µL of 2 × SYBR Green PCR Master Mix, 0.4 µL of each forward and reverse primer (10 µM), 1 µL of 10-fold diluted template DNA, 8.2 µL of RNase-free ddH_2O	1.5 min at 95 °C, followed by 40 cycles of 30 s at 95 °C, 45 s at 64°C, and 45 s at 72 °C. Data collection at 72 °C	280 bp
	Fg16N-R	TTCTTTGACATCTGTTCAACCCA			

Table S4. Environmental residual concentrations of pesticides and one and ten times the pesticide application concentrations.

Pesticides	Environmental residual concentration range (mg kg^{-1})	Application concentrations (mg kg^{-1})	Ten times of the application concentrations (mg kg^{-1})
Epoxiconazole	0.010-0.620	0.650	6.50
Hexaconazole	0.001-0.470	0.500	5.00
Imidacloprid	0.010-0.538	0.550	5.50
Isoproturon	0.014-0.053	0.050	0.500
Mesosulfuron-methyl	0.001-0.065	0.065	0.650

Table S5. The standard curve reaction system of the detection method of H_2O_2.

	1	2	3	4	5	6	7
10 mM H_3PO_4	0.5 mL	0.5 mL	0.5 mL	0.5 mL	0.5 mL	0.5 mL	0.5 mL
1 M KI	1 mL	1 mL	1 mL	1 mL	1 mL	1 mL	1 mL
0.5 mL H_2O_2	0.00 mg/L	0.05 mg/L	0.10 mg/L	0.50 mg/L	1.00 mg/L	5.00 mg/L	10.00 mg/L
10 mM (NH)MoO	0.1 mL	0.1 mL	0.1 mL	0.1 mL	0.1 mL	0.1 mL	0.1 mL

Table S6. UPLC–MS/MS conditions of the analytes studied.

Compounds	Ion source	CV (V)	Quantification ion transition	CE1 (eV)	Confirmatory ion transition	CE2 (eV)
DON	ESI+	150	297.2→249.1	5	297.2→203	15
Epoxiconazole	ESI+	120	330→141	20	330→121	20
Imidacloprid	ESI+	92	256.1→209.1	9	256.1→175.1	17

Table S7. Recoveries and RSD values for DON, epoxiconazole and imidacloprid in two spiked levels from different matrices.

Compound	Matrix	Spiked level (mg kg^{-1})	Recovery (%,n=5)	RSD[a] (%,n=5)
DON	TBI medium	0.01	88.3	9.50
		1	94.3	4.50
	Wheat leaf	0.01	82.5	3.50
		1	106.9	7.80
	Wheat ear	0.01	94.6	12.1
		1	97.4	3.50
Epoxiconazole	Wheat leaf	0.01	102.3	6.30
		1	97.4	5.10
	Wheat ear	0.01	111.4	6.90
		1	96.1	5.70
Imidacloprid	Wheat leaf	0.01	86.5	3.20
		10	84.9	1.50
	Wheat ear	0.01	90.6	5.40
		10	102.0	1.60

[a]RSD, the relative standard deviation
